# Quantitative stain-free imaging and digital profiling of collagen structure reveal diverse survival of triple negative breast cancer patients

**DOI:** 10.1186/s13058-020-01282-x

**Published:** 2020-05-06

**Authors:** Laurent Gole, Joe Yeong, Jeffrey Chun Tatt Lim, Kok Haur Ong, Hao Han, Aye Aye Thike, Yong Cheng Poh, Sidney Yee, Jabed Iqbal, Wanjin Hong, Bernett Lee, Weimiao Yu, Puay Hoon Tan

**Affiliations:** 1 Institute of Molecule and Cell Biology, A*STAR, 61 Biopolis Drive, Proteos, Building, Singapore, 138673 Singapore; 2 Department of Anatomical Pathology, Singapore General Hospital, Singapore, Singapore; 3 Singapore Immunology Network, A*STAR, 8A Biomedical Grove, Immunos Building, Biopolis, Singapore, 138648 Singapore; 4 Department of Pathology, National University Hospital, Singapore, Singapore; 5 Diagnostic Development Hub (DxD), A*STAR, Singapore, Singapore; 6 Division of Pathology, Singapore General Hospital, 20 College Road, Academia, Level 7, Diagnostics Tower, Singapore, 169856 Singapore

**Keywords:** Triple-negative breast cancers, Collagen profile, Quantitative imaging, Second harmonic generation microscopy, Stroma

## Abstract

**Background:**

Stromal and collagen biology has a significant impact on tumorigenesis and metastasis. Collagen is a major structural extracellular matrix component in breast cancer, but its role in cancer progression is the subject of historical debate. Collagen may represent a protective layer that prevents cancer cell migration, while increased stromal collagen has been demonstrated to facilitate breast cancer metastasis.

**Methods:**

Stromal remodeling is characterized by collagen fiber restructuring and realignment in stromal and tumoral areas. The patients in our study were diagnosed with triple-negative breast cancer in Singapore General Hospital from 2003 to 2015. We designed novel image processing and quantification pipelines to profile collagen structures using numerical imaging parameters. Our solution differentiated the collagen into two distinct modes: aggregated thick collagen (ATC) and dispersed thin collagen (DTC).

**Results:**

Extracted parameters were significantly associated with bigger tumor size and DCIS association. Of numerical parameters, ATC collagen fiber density (CFD) and DTC collagen fiber length (CFL) were of significant prognostic value for disease-free survival and overall survival for the TNBC patient cohort. Using these two parameters, we built a predictive model to stratify the patients into four groups.

**Conclusions:**

Our study provides a novel insight for the quantitation of collagen in the tumor microenvironment and will help predict clinical outcomes for TNBC patients. The identified collagen parameters, ATC CFD and DTC CFL, represent a new direction for clinical prognosis and precision medicine. We also compared our result with benign samples and DICS samples to get novel insight about the TNBC heterogeneity. The improved understanding of collagen compartment of TNBC may provide insights into novel targets for better patient stratification and treatment.

## Background

Breast cancer is the second leading cause of cancer-associated mortality in women [1] and represents a serious global health concern. WHO has estimated that globally, > 1.7 million new breast cancer cases and 522,000 breast cancer-associated deaths occurred in 2012 [1]. Breast cancer can be classified as either non-invasive ductal carcinoma in situ (DCIS) or invasive ductal carcinoma (IDC). Invasive tumors can be further categorized into different molecular subtypes, including luminal A, luminal B, Her2^+^, and basal-like/triple-negative breast cancers (TNBCs), based on expression profiling or protein surrogates [2–4]. TNBC is defined as ER^(−)^, PR^(−)^, and HER2^(−)^ and is typically more heterogeneous and aggressive than other subtypes, resulting in a relatively poor prognosis. Scant data and lack of biomarkers limit us to build robust models that can stratify patients and predict clinical outcomes [5–9]. Differences between ethnic groups and uneven reporting serve to further complicate matters. For example, while the metabolic profile of African-American TBNC patients has been reported [10], Asian women are subjected to different risk factors due to lifestyle and genetic differences, which can modulate treatment responses and impact disease outcomes [11–16]. Despite the association between breast stromal collagen density and invasive breast cancer, which is second only to deleterious germline BRCA1 and BRCA2 mutations [17, 18], stromal biology remains poorly delineated compared with other cancer cell compartments and immune cell populations. Although some previous breast cancer studies incorporate Asian women, large-scale, in-depth, and specific collagen structural data from non-Caucasian women, especially those with TNBC, are lacking. In this work, our group sought to investigate the effect of collagen structure on TNBC outcomes in a cohort of Asian women.

The PD-L1/PD-1 treatment is promising, while the overall response rate is not ideal including breast cancer. Many factors may impact the patient outcome. Mesenchymal cells, immune cells, extracellular matrix (ECM) components, lymphatics, and vasculature are all present in breast cancer stroma. Immune cell infiltration, disruption of the basal membrane and myoepithelial cell layer, and remodeling of stromal collagen are among the earliest key events in the development of invasive breast cancer [19, 20]. Recent studies have also demonstrated that stromal reaction influences the efficacy of particular treatments, including immunotherapy [21, 22]. Furthermore, the importance of stromal and collagen biology in breast tumor progression is highlighted by differential immune, angiogenic, and fibroblastic responses, and an array of stromal genes may be used to predict the clinical outcome [23, 24]. Collagen fibers represent the major structural ECM component in breast tumors, and increases in stromal collagen fibers have been demonstrated to facilitate breast tumor formation, invasion, and metastasis. Collagen is secreted by cancer-associated fibroblasts (CAFs), which are involved in tumor stromal activation, and may lead to tumor progression through multiple mechanisms, including neoangiogenesis, tumor cell proliferation, and invasion [25–29]. CAFs also affect tumor progression by reprogramming the tumor microenvironment at both the metabolic and immune levels and by promoting adaptive resistance to chemotherapy [30]. In this work, we only focus on the quantification of the collagen remodeling and explore its impact on the TNBC patient survival.

Stromal collagen remodeling is characterized by collagen realignment in the stromal compartment, and multiple recent studies have demonstrated that collagen structure, profiles, and patterns within the tumor stromal microenvironment have diagnostic and prognostic value [31–40]. However, the role of collagen remodeling in tumor formation and metastasis is complex, and while correlations between breast tissue density and tumor formation have been reported [31], the relationships between collagen density, structure, and cancer cell migration and invasion remain to be fully understood. Collagen in breast tissue was historically thought to form a physical barrier that prevents tumor cell migration, with collagen degradation and deposition being prerequisites for this process [31, 33, 34, 38, 39]. Furthermore, three tumor-associated collagen signatures (TACs) have been reported [40], and radial alignment of collagen fibers around the boundaries of tumors is associated with tumor cell invasion. As a result, this topic remains the subject of debate within the field, and no conclusive statement has been reached.

This issue may, at least partially, be due to historical methodological limitations. The remodeling and quantification of collagen have traditionally been studied using biochemical staining techniques, such as Picrosirius Red (PSR) staining or Masson’s trichrome (MT) staining. However, this analysis is highly dependent on the staining protocol and color deconvolution pipeline in digital image analysis. Due to the limitations presented by these imaging techniques, only the total amount of collagen present in tissue samples is typically assessed, with the quantitative structure of collagen fibers remaining beyond their scope. However, the development of quantitative stain-free collagen imaging techniques, such as second harmonic generation (SHG), permits the quantification of collagen structure at a finer level of detail. In this study, we used two-photon excitation (TPE) and SHG to scan breast cancer tissue microarrays (TMAs). SHG is a multiphoton, laser-based, quantitative non-linear optical imaging technique used to identify fibrillary collagen in fixed tissues. Due to its physical principles, it is highly sensitive to changes in collagen fibril and fiber structure and also to the remodeling of connective tissue.

We developed a fully automatic digital collagen profiling platform based on TPE/SHG imaging techniques to quantify collagen using four parameter categories: intensity/area, textural, structural, and fiber distribution features. Numerical image features were extracted from each image. The extracted features were selected using feature selection algorithms, and associations between these features and the existing clinicopathological parameters were investigated across the whole patient cohort. Bioinformatics models were designed for the purpose of classification (diagnosis) and prediction (prognosis). Our results provide a computational solution to classify collagen into two distinct modes based on fiber SHG signal intensity, texture, and morphology: aggregated thick collagen (ATC) and dispersed thin collagen (DTC). Several imaging features were strongly correlated with clinicopathological characteristics, and ATC collagen fiber density (CFD) and DTC collagen fiber length (CFL) were revealed to be of prognostic value based on our patient cohort and their clinical outcomes. Separation of ATC and DTC provides a novel understanding of collagen remodeling during cancer progression, and our results may help to resolve the debate over whether collagen has a role in inhibiting or promoting patient survival. All extracted parameters are listed in Supp. Table 0^1^, and they are quantified in ATC and DTC region separately.

## Materials and methods

### Tissue samples

Formalin-fixed paraffin-embedded (FFPE) human breast cancer tissues were obtained from the Department of Anatomical Pathology, Division of Pathology, Singapore General Hospital (SGH). The present study utilized archival specimens from a cohort of 388 TNBC breast cancer patients diagnosed at the Division of Pathology of SGH from 2003 to 2015. The Centralized Institutional Review Board (IRB) of SingHealth provided ethical approval for the use of patient material in this study (CIRB ref: 2018/2910-2013/664/F). Clinicopathological parameters including age, ethnicity, tumor size, histologic grade, subtype, associated ductal carcinoma in situ, lymphovascular invasion, axillary lymph node status, tumor borders, and growth patterns were reviewed.

The tissues were fixed and processed in Surgipath 10% neutral buffered formalin (Leica Biosystems, Wetzlar, Germany) for 6–48 h in accordance with the American Society of Clinical Oncology/College of American Pathologists (ASCO/CAP) Clinical Practice Guidelines [41, 42].

### TMA construction

Hematoxylin and eosin (H&E)-stained tumor sample slides were reviewed. Three tumor cores (1 mm diameter) were punched from corresponding donor FFPE blocks. The three cores are reviewed by clinical pathologists, and the selected representative core is used to construct the TMA assays using an MTA-1 Manual Tissue Arrayer (Beecher Instruments, Inc., Sun Prairie, WI, USA), as previously described [43].

### Image acquisition with SHG

The constructed TMA assays were deparaffinized and then scanned using the Genesis 200™ system (HistoIndex Pte. Ltd., Singapore). The laser power of the system was set at “High,” and binning was set at two frames to reduce the noise level. The brightness of each pixel represents the collagen density in the fiber.

### Image analysis

A total of eight TMAs was scanned, and corresponding images were acquired, containing data from all 388 TNBC patients. These images were then processed according to the computational pipeline jointly developed by the pathologists and computer scientists. The details of this pipeline are described later. Each image of a given TMA sample was named according to its pathological identification, linking the given image to clinicopathological parameters.

**Fig. 1:**
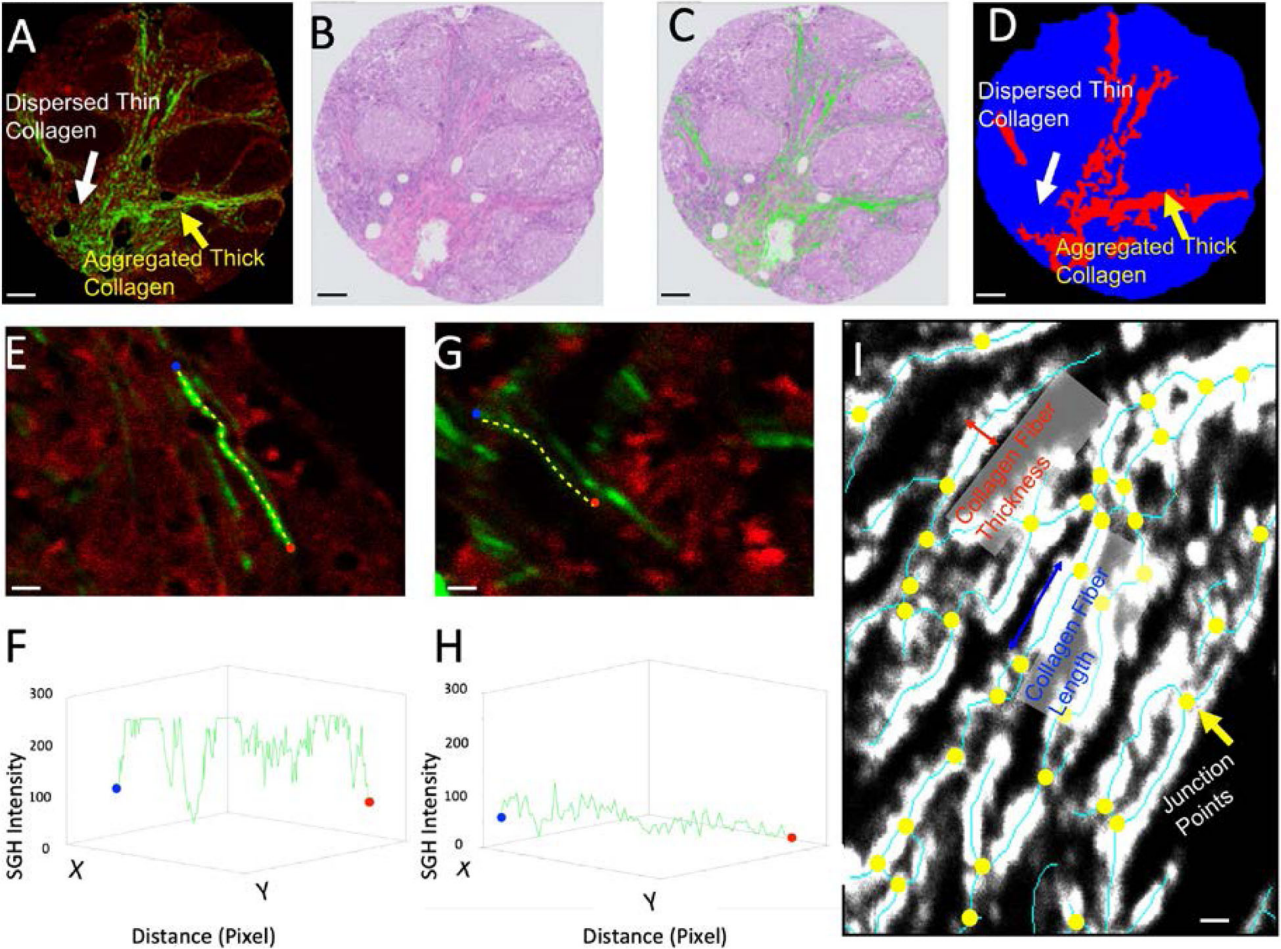
TPE/SHG technique and digital collagen structural profiling for TNBC TMA samples. **a** TPE/SHG image of TNBC TMA. The red channel is the TPE channel of excitation wavelength 780 nm and emission wavelength 560 nm; the green channel is the SHG channel (wavelength 380 nm), known as second harmonic or frequency doubling. SHG is highly sensitive to changes in collagen fibril and fiber structures. **b** A representative patient TMA sample stained by H&E. One unique advantage of the TPE/SHG imaging technique is that it is stain free. Once the SHG image is acquired, the same sample can be stained with H&E and scanned as a digital image. **c** The overlay of the collagen structure acquired in SHG and the H&E images. Since **a** and **b** are two images of the same physical slide, it is possible to fuse them together. **d** The differentiation of tissue area occupied by aggregated thick collagen (ATC) and dispersed thin collagen (DTC) based on intensity, texture, and morphology. The red area, as shown by the yellow arrow in **a** and **d**, is the ATC area; the blue area, shown by the white arrow in **a** and **d**, is the DTC area. The ATC area primarily contains aggregated collagen, and the DTC area contains dispersed collagen. Some parts of the DTC area may contain relatively small collagen compartments. **e** A brighter collagen fiber and **f** its SHG intensity reading. **g** A weaker collagen fiber and **h** its SHG intensity reading. The measurements are taken starting from the blue dot and ending at the red dot along the path of the yellow dotted line. Collagen fiber density (CFD) is a unique quantitative reading from SHG which indicates the collagen packing structure. **i** Once the fiber is segmented using the Gaussian mixture model (GMM), the structural information of the collagen can be extracted. The yellow dots are the junction points. It is then possible to measure different features, including CFL, CFT, and CRI. Scale bars: **a**–**d** 100 um, **e**, **g**, **i** 2 um

A representative TNBC TMA image is presented in Fig. 1a, including the TPE/SHG image and the H&E-stained image in Fig. 1b. After a TPE/SHG image has been taken from the unstained slides, the same slide can be stained using H&E. The collagen compartments can then be merged with the H&E image, as shown in Fig. 1c. The merging of collagen with H&E provides information concerning collagen remodeling at different regions in the tissue. Although PSR or MT staining is often used to quantify collagen in tissue samples, these are not truly quantitative imaging solutions, as the red color in PSR and the blue color in MT staining do not necessarily indicate collagen fiber structure. SHG, however, is a laser-based quantitative imaging technique. Once the system is well calibrated, the physical principles result in a numerical reading of collagen density, as shown in Fig. 1e–h. Fiber density is a critical indication of the collagen packing structure. A representative stronger and weaker fiber signal is shown in Fig. 1e, g; their respective SHG signal intensity is shown in Fig. 1f, h. Measurements of SHG intensity are taken starting from the blue dot and ending at the red dot along the path of the yellow dotted line. A brighter collagen fiber has much higher SHG intensity in Fig. 1f, and a weaker collagen fiber has lower SHG intensity. Besides the collagen fiber density, named as CFD in this work, it is possible to extract other collagen structural information from the images.

The processing of the TMA image is as follows: A sample mask was first defined to cover the area occupied by the TMA tissue. Within this given region, a two-mode Gaussian mixture model (GMM) was applied to determine whether a given pixel signaled positive for collagen. Based on the GMM model, an optimized thresholding value was determined to binarize the image, creating a collagen binary image. A similar scheme was applied to the red channel to create a tissue binary image. The collagen area ratio (CAR) was then measured based on the area of the collagen binary image and sample mask. The collagen fiber density (CFD) is the ratio between the summation of the total collagen density signal within the collagen binary image and the area of the collagen binary image. Skeletonization was applied to the collagen binary image in order to identify the central line of the collagen network, as shown by the red solid line in Fig. 1i. The junction points were then identified, as shown by the yellow dots in Fig. 1i. The number of junction points is an indication of the complexity of the collagen network. The ratio between the number of junction points and the total length of the skeleton provides the collagen reticular index (CRI). Other structural features, such as collagen fiber length (CFL) and collagen fiber thickness (CFT), were also measured accordingly. A list of key parameters and their definitions are provided in Supp. Table 0^2^. All parameters in this work are measured at the ATC, DTC, and ALL tissue regions, which is the combination of both ATC and DTC.

In order to separate ATC and DTC in the tissue, an intensity and morphology filtering pipeline was developed. Based on the intensity and morphology (the degree of collagen aggregation), it is possible to differentiate the sample mask into two distinct modes, as presented in Fig. 1d. We applied an intensity morphological opening operation with a diameter bigger than the 3 times of collagen fiber thickness to identify the aggregated collagen area, i.e., ATC area which is red in Fig. 1d. The rest of the sample mask area, i.e., blue area in Fig. 1d, will be defined as the DTC area. We also tested the diameter for the opening operation, and it is robust to identify isolated fibers. Intensity/area, textural, structural, and fiber distribution features were measured separately for both ATC and DTC. Once the features were extracted from the images, their relationships with clinicopathological parameters were assessed. Associations with low *p* values are presented in corresponding figures. The selected features were then used to build a prediction model for patient survival.

### Statistical analysis pipeline

Follow-up data were obtained from medical records for the entire cohort. Disease-free survival (DFS) and overall survival (OS) were defined as time from the point of diagnosis to recurrence or to death/the date of the last follow-up, respectively. Statistical analysis was performed using the R statistical language (version 3.3.1). The relationships between clinicopathological parameters and collagen profiles were tested using *χ*^2^ and Fisher’s exact tests. Survival analyses were conducted using univariate Cox regression models based on binary thresholded imaging parameters. The final threshold was selected from a range of thresholds determined using the percentiles of the imaging parameters in 10% increments that gave the best separation between the two groups.

## Results

### Numerical collagen structural parameters are associated with clinicopathological parameters

Numerical collagen features were extracted from digital images as described in the “Materials and methods” section. Detailed formulations and definitions of the key parameters are provided in Supp. Table 0^2^. The association between numerical imaging features and clinicopathological parameters was studied. All defined features are measured separately in the ATC area (red area in Fig. 1d), DTC area (blue area in Fig. 1d), and ALL area (the combined red and blue area in Fig. 1d). For example, ATC CFD, DTC CFD, and ALL CFD were measured as different regions for CFD.

**Fig. 2:**
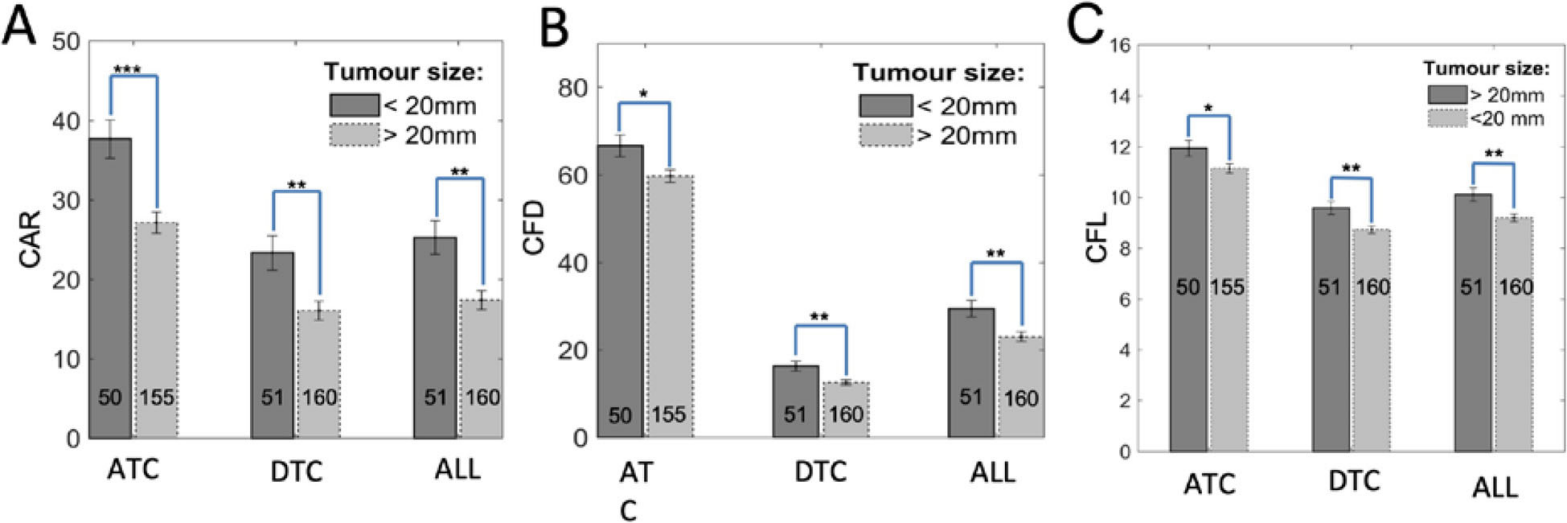
The extracted numerical collagen structural parameters are associated with tumor size. **a** ATC and DTC CAR are significantly decreased in the samples of tumor size > 20 mm compared with ≤ 20 mm. **b** ATC and DTC CFD are higher in ≤ 20 mm samples compared with > 20 mm. **c** ATC and DTC CFL are longer in ≤ 20 mm samples compared with > 20 mm

Tumor size is a key clinicopathological parameter used to evaluate the likelihood of cancer progression. We screened the possible associations between our numerical collagen features and tumor size. As shown in Fig. 2a, there are significant differences in both ATC CAR and DTC CAR between patients with tumors of ≤ 20 mm and > 20 mm. (Note that the significant difference in ALL CAR in Fig. 2a is a weighted measurement between ATC and DTC.) The ATC area ratio, which represents the ratio between the red area and the whole TMA area as shown in Fig. 1d, was also higher of tumor > 20 mm, but no significance (data not presented). Notably, there was a significant difference in ATC CFD, DTC CFD, and ALL CFD between patients with tumors of ≤ 20 mm and > 20 mm in Fig. 2b. This is a strong indication that larger tumors tended to have a stronger impact in microenvironment remodeling around tumor nests. Furthermore, there was a significant difference in both ATC CFL and DTC CFL in Fig. 2c. The latter points towards an interplay between tumor size and the surrounding stromal environment remodeling.

**Fig. 3:**
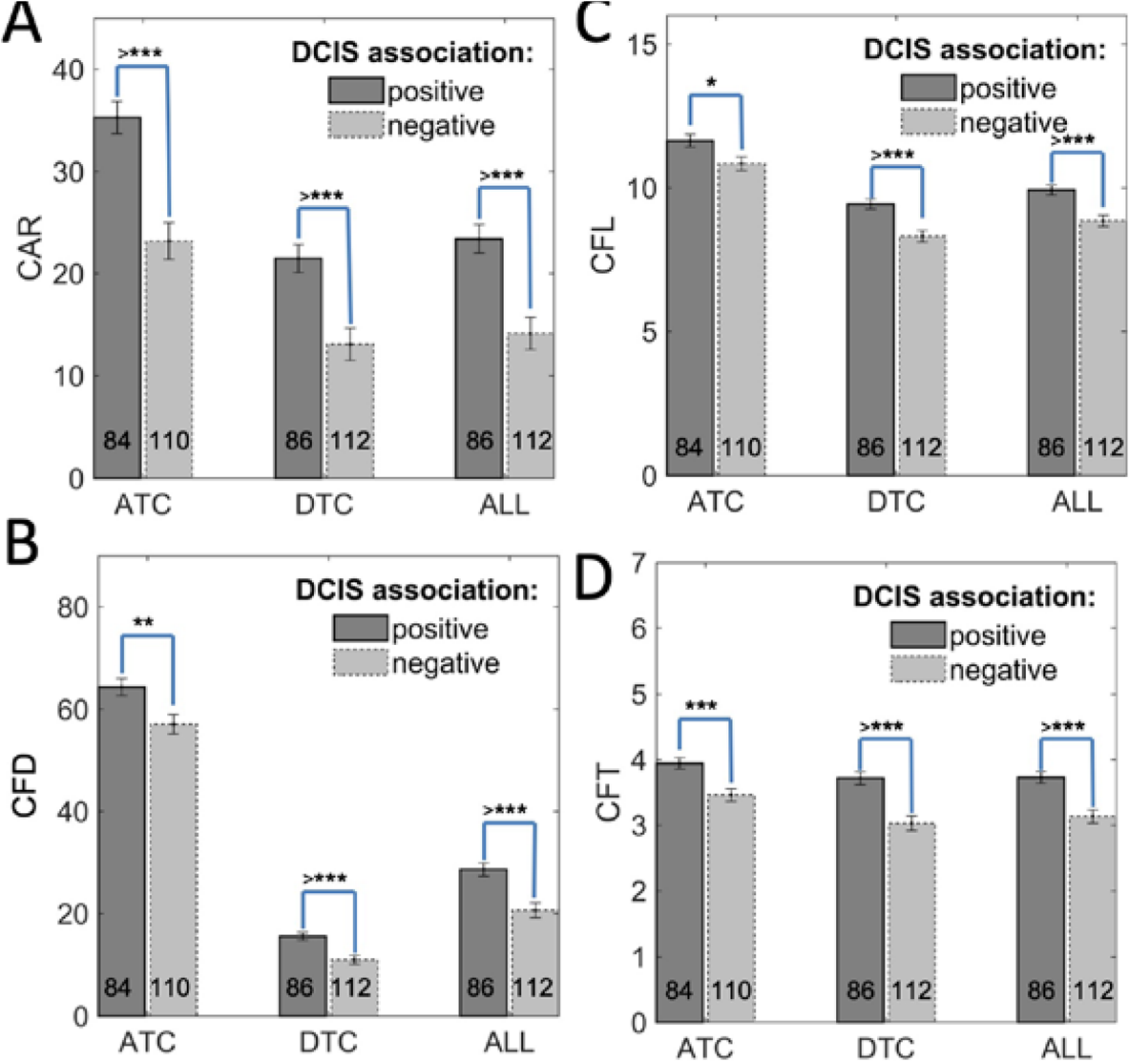
The extracted numerical collagen structural parameters are relevant with DCIS association. **a** ATC and DTC CAR are significantly higher in patients with positive DCIS association compared with those with no DCIS association. **b** ATC and DTC CFD are increased in patient samples with positive DCIS association. The CAR and CFD results indicate that the collagen may form a stronger protective barrier surrounding the tumor nests. **c** DTC CFL significantly increases for patients with DCIS associations. **d** ATC CFT and DTC CFT significantly differed between patients with DICS associations and those with no DCIS associations. Error bars represent SE of the mean. *p < 0.05, **p < 0.0, ***p < 0.001, and ^>^***p < 0.0001, with comparisons indicated by lines. *p* values calculated using one-way ANOVA and Tukey’s HSD test

The correlation between the quantitative collagen features and the degree of DCIS association for those TNBC patients were of particular interest. The DCIS association is a pathological feature to describe if the TNBC patient is associated with DICS subtype. First, patients with associated DCIS had a greater ATC area ratio compared with those with no associated DCIS. ATC CAR and DTC CAR were also significantly higher in patients with a positive DCIS association (Fig. 3a). The data indicated that ALL CAR was significantly different in these two sub groups. ATC CFD was higher in patient samples with associated DCIS compared with those with no associations (Fig. 3b). This indicated that ATC collagen may have formed a stronger protective barrier surrounding the DCIS. There was a strong significant difference in DTC CFD (Fig. 3b) and DTC CFL (Fig. 3c), as well as in ATC CFT and DTC CFT (Fig. 3d) between patients with associated DCIS and those with no associated DCIS. This may imply that TNBCs which harbored associated DCIS potentially have different fiber packing structures, which may cause differences in fiber brightness, length, and thickness. The aggregation of the collagen is relevant with DCIS association.

Certainly, not all clinicopathological parameters are relevant with the extracted quantitative features, which describe the properties of collagen. For example, tumor grade is a critical pathological parameter associated with tumor behavior and is evaluated based on nuclear pleomorphism, mitotic activity, and tubule formation. However, we did not find a strong association between our numerical collagen parameters and tumor grades in our patient cohort. One potential rational is that although tumor grading is a key feature used in prognostication, it is not directly linked with collagen remodeling. The CFL and CFD results are presented in Supp. Figure 1A and Supp. Fig. 1B, respectively.

**Fig. 4:**
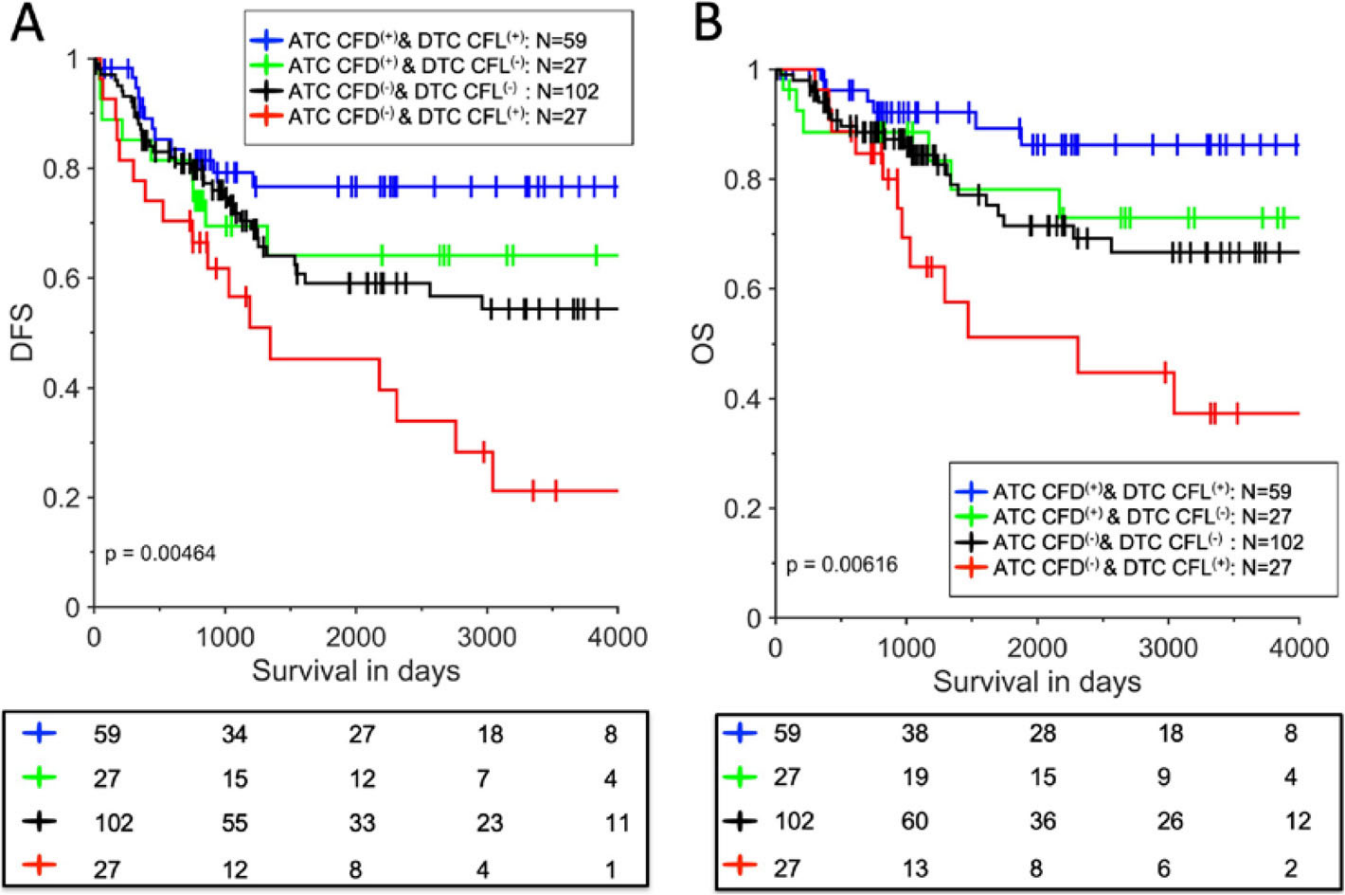
The Kaplan-Meier survival curves of a two-parameter prognostic model to stratify the TNBC patients. **a** The two-parameter model of ATC CFD and DTC CFL for DFS prediction. **b** The two-parameter model of ATC CFD and DTC CFL for OS prediction. Our data demonstrates the heterogeneity of the TNBC subtype. The number of patients in each group is also moderately well balanced. The blue curve represents ATC CFD^(+)^ and DTC CFL^(+)^, the red curve represents ATC CFD^(−)^ and DTC CFL^(+)^, and the two other conditions are represented by the black and green curves

In order to evaluate the prognostic value of the collagen parameters, we performed univariate Cox regression univariate analyses. Most of the parameters listed in Supp. Table 02 are able to stratify the patients in their own way. For example, we found out that “higher” ATC CFD was associated with improved DFS and OS in TNBC patients (DFS, *p* = 0.0163; OS, *p* = 0.0085). All cutoff values of the parameters were determined as described in the “Materials and methods” section. The Kaplan-Meier survival plots for ATC CFD are shown in Supp. Figure 2A-B. We further explore if any combinations of two quantitative collagen parameters are able to stratify the patients in more detail. *We found ATC CFD combined with DTC CFL was of significant prognostic value for patient DFS and OS.* (The survival curves of DTC CFL are shown in Supp Fig. 2C-D (DFS, *p* = 0.3; OS, *p* = 0.24)). Predictive prognostic models are essential for clinical practice to stratify the patients in the healthcare system. Figure 4a, b indicates that when the 177 patients were divided into four distinct groups based on ATC CFD and DTC CFL, they experienced significant differences in survival (DFS, *p* = 0.0046; OS, *p* = 0.0062). This further demonstrates the heterogeneity of the TNBC subtype. The number of patients in each group is also moderately well balanced (Fig. 4). “Brighter” ATC CFD and “longer” DTC CFL indicate the best prognosis, as shown by the blue curve, while “weaker” ATC CFD and “longer” DTC CFL indicate the worst, as shown by the red curve. The other two conditions, represented by the black and green curves, fall between the aforementioned extremes and result in intermediate patient survival. The impacts of different significant parameters on these four groups of patients are summarized in Supp. Table 3.

**Fig. 5:**
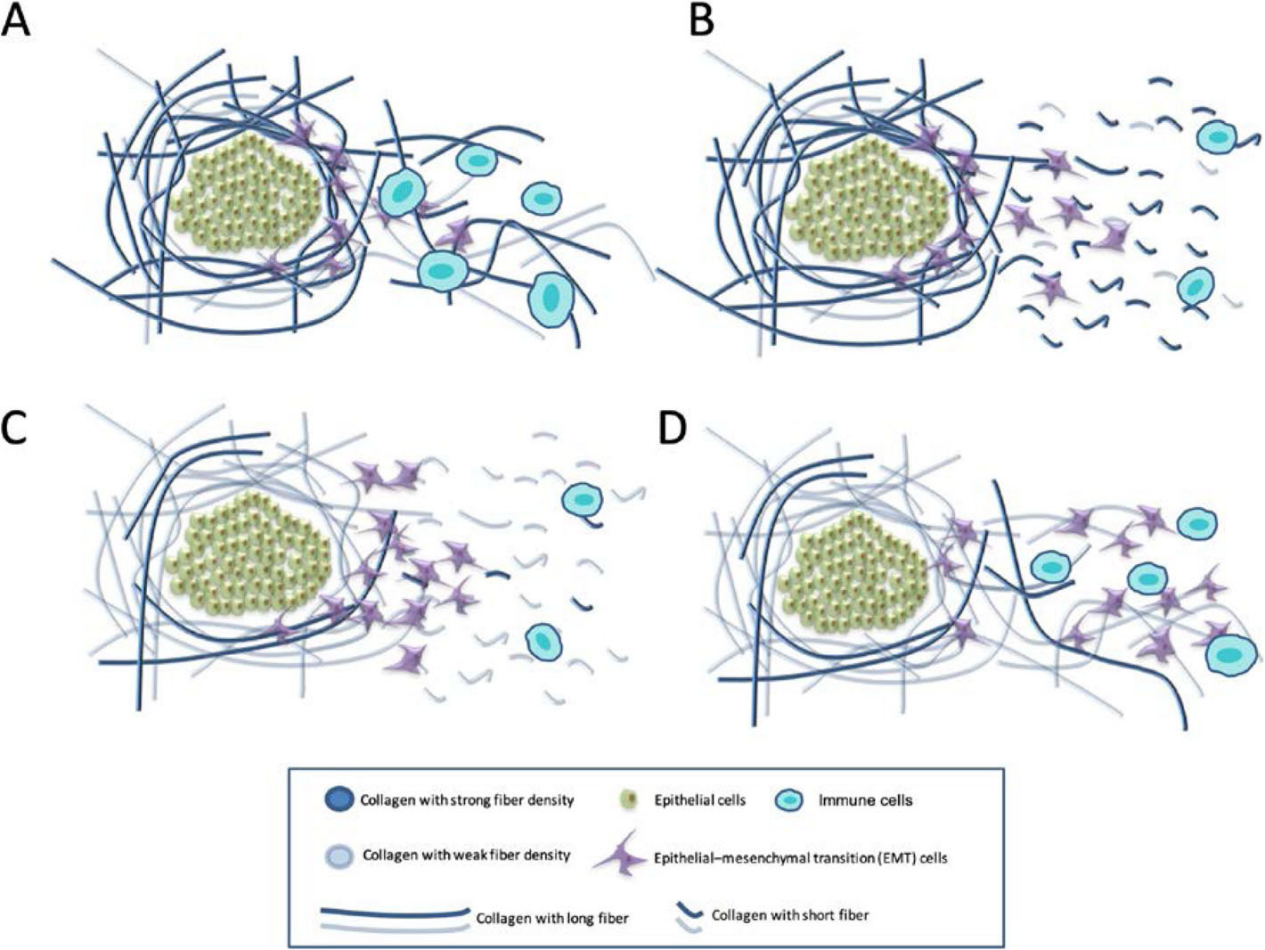
The mechanism of our two-parameter prognostic model using collagen structural information. **a** ATC CFD^(+)^ indicates the presence of a strong layer of collagen fibers around the tumor nests, and DTC CFL^(+)^ indicates longer collagen fibers. Only a few cancer cells escape; the strong layer of collagen fibers and the longer collagen fibers help the immune cells deal with the malignant cells. **b** Although cancer cells are less able to escape from the tumor nest in this condition, DTC CFL^(−)^ status suggests that the fiber is relatively short and makes it more difficult for the immune cells to respond to potential malignant cell invasion. **c** ATC CFD^(−)^ indicates a weaker protective collagen layer. In this case, cancer cells may easily escape the tumor nest. Although the collagen fibers are shorter in this condition, i.e., DTC CFL^(−)^, the larger number of cancer cells escaping may cause a higher chance of metastasis. **d** These tumors have a weaker protective collagen layer around the tumor nests and have long DTC collagen fibers. Cancer cells may both escape with relative ease and use the longer DTC collagen fiber to migrate more efficiently overwhelming the immune cell response; hence, the stromal collagen fiber length plays a bipartite role—being beneficial to the immune cells as long as the ATC CFD stays high, or conversely becoming beneficial to malignant cells when this protective layer of collagen weakens. This resulted in the worst survival among the four groups

Our results may help resolve the historical dilemma concerning the role of collagen in breast cancer. Proposed mechanisms underlying the effect of ATC CFD and DTC CFL on survival are presented in Fig. 5a–d. The differences may be due to the collagen bonds aggregating in different ways: for example, ATC CFD^(+)^ represents a protective structure formed by the strongly aggregated collagen. Depending on how strong this first protective structure is, we hypothesize that DTC CFL^(+)^ may represent a potential “highway” that facilitates either immune cell migration or cancer cell migration. As presented in Fig. 5a, ATC CFD^(+)^ may manifest as a strong layer of collagen fibers around the tumor nest. It would potentially be difficult for malignant cells to penetrate this layer and escape from their original site. Even when cells escape, in the DTC CFL^(+)^ condition, long collagen fibers facilitate migration of immune cells to respond and deal with malignant cells. This combination, therefore, provides the best survival outcome for the patients as illustrated in Fig. 5a, with cancer cell migration potentially inhibited. Figure 4d, however, shows the opposite extreme. In the ATC CFD^(−)^ condition, the collagen may be unable to form a strong protective barrier, meaning that cancer cells are able to escape easily. Once this occurs, longer collagen fibers in the DTC area (DTC CFL^(+)^) may facilitate their migration more efficiently and therefore overcome the capacity of immune cells to respond to the invasion. Figure 4b, c shows the two intermediate conditions. In this proposed model, patient survival is primarily impacted by the following two factors: (i) how easily the malignant cells are able to escape from their original site (This is relevant with the ATC CFD and ATC area ratio.) and (ii) how easily they can migrate once this occurs, which is associated with the DTC CFL. We show here how the stromal collagen can have a bipartite role in cancer progression promoting either malignant cell migration or immune cell response depending on the state of the ATC CFD and DTC CFL.

**Fig. 6:**
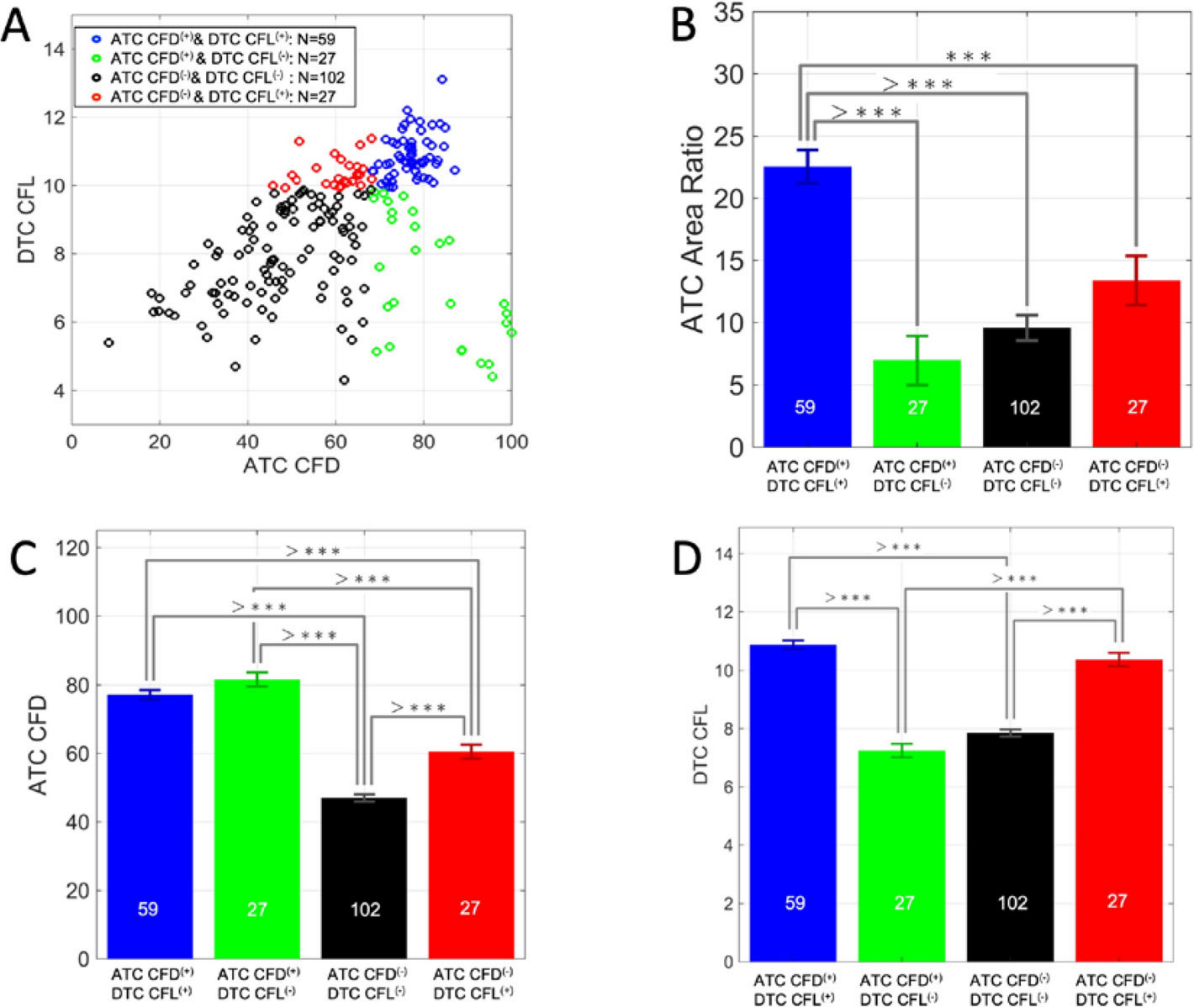
Key collagen structural differences between the four groups of patients. **a** The distribution of the patients in the two-parameter space. **b** ATC area ratio, which is defined as the ratio between the aggregated collagen region area (the red area in Fig. 1d) and the total tissue area (the combination of the red and blue areas in Fig. 1d), is one of the key aspects of patient survival. This indicates the percentage of the given tissue area that is occupied by ATC. Patients with the best survival had significantly higher ATC area ratios compared with the other groups. **c**, **d** Differences in ATC CFD and DTC CFL in the four different groups, respectively. Error bars represent SE of the mean. *p < 0.05, **p < 0.0, ***p < 0.001, and ^>^***p < 0.0001, with comparisons indicated by lines. *p* values calculated using one-way ANOVA and Tukey’s HSD test

The 2D distribution of patient ATC CFD and DTC CFL is presented in Fig. 6a. The color-coded spots indicate the patient groups in Fig. 4. However, the mechanism underlying collagen aggregation remains unclear. The ATC area ratio is defined as the ratio between the red area in Fig. 1d and the total tissue area (the combination of red and blue areas in Fig. 1d). This means the percentage of the given tissue area that is occupied by ATC. The ATC area ratio of patients with the best survival, i.e., ATC CFD^(+)^ and DTC CFL^(+)^ patients presented by the blue survival curve in Fig. 3a, b, was significantly higher than in the other three conditions as shown in Fig. 6b. Even if we only compared with ATC CFD^(+)^ and DTC CFL^(−)^ patients, represented by the green bar in Fig. 6b, the ATC area ratio of patients with the best survival was almost threefold higher. Based on our patient grouping, the ATC CFD of the patients with the best survival is also significantly higher than the group with the worse survival. The following two conditions were therefore associated with optimal survival: (i) an increased amount of aggregated collagen in the tissue and (ii) brighter collagen fibers, which provide indirect evidence of different collagen fiber microstructures. These results provide evidence suggesting that collagen aggregation itself plays a key role in patient survival. Figure 6d shows the DTC CFL for the four patient groups. Differences in DTC may cause different degrees of cell migration, including both immune cells and malignant cells, in the tissue, as well as subsequent invasion, with longer collagen fibers facilitating cancer cell migration to remote areas. Thus, collagen fiber length may also have impacted patient survival.

## Conclusion and discussion

Increased computational power and the integration of computational solutions with artificial intelligence (AI) and machine learning (ML) have resulted in substantial improvements to clinical diagnosis and prognosis. This has had significant implications for the field of histopathology too, where traditional assessments typically involve qualitative characterization on a numerical basis. The resultant discrete numbers are often not sufficient to build robust mathematical models for clinical diagnosis or prognosis, particularly when generated from manual assessments with certain subjective inconsistency. Thus, scanning and digitization of glass slides represent the future for AI-assisted pathology. Imaging techniques are also changing, shifting from basic H&E staining to other techniques, including immunohistochemistry, immunofluorescence, and stain-free imaging techniques, such as SHG. In this work, we applied TPE/SHG to investigate the effect of collagen structures on the prognosis of TNBC patients, which is an aggressive but heterogeneous subtype. SHG imaging quantified the collagen component, including collagen types I and III. Our team developed an automatic image analysis solution to extract different collagen features from the SHG images, including collagen structural information.

The literature has reported that while collagen can potentially form a protective barrier and prevent cancer cells from escaping their original site, collagen fibers have also been found to serve as a “highway” that facilitates cancer cell migration to remote locations, impacting patient survival [44–47]. Previously, the mechanisms underlying these contradictory reports were unclear [48]. This historical lack of understanding was due to the paucity of methods to differentiate between collagen structures. As demonstrated by a number of previous studies, it is an oversimplification to characterize collagen remodeling as a simple increase or decrease in total ECM collagen content [48]. It is more important to understand the structural differences in various regions under diverse conditions. For example, the orientation of collagen fibers, such as TACs, is a key parameter [40]. Fibrils are packed to form collagen fibers. The organization and distribution of these fibers within the tissue are also important, for example, whether the collagen forms any aggregations. One unique parameter generated by SHG in the present study was CFD, which provides a strong indication of collagen fiber strength and fibril packing structure, as the laser power and system calibration generate a correlation between pixel brightness and collagen strength. In our study, we showed some extracted parameters were strongly associated with certain clinicopathological features, including tumor size and DCIS associations. However, tumor grade was not relevant with the investigated collagen features as we expected. This is because grade assessment is mainly based on nuclei morphological and packing features. It is not linked to collagen characteristics. Regardless, the results of our study suggest the importance of assessing collagen structure, as it appears to represent a potential key prognostic parameter. According to our data, tumor grade and tumor size are also poor predictors of prognosis, as shown in Supp Fig. 3.

Furthermore, our results suggest that the collagen compartment has two distinct modes, named ATC and DTC, and differences between these modes underpin the different roles of collagen. Collagen remodeling can either prevent or promote cancer cell migration, and while the mechanism remains to be fully understood, we demonstrated that ATC CFD and DTC CFL together have strong prognostic value. Higher ATC CDF indicates the presence of stronger collagen fibers surrounding the tumor nest, through which it is difficult for cancer cells to escape (Fig. 5a). Therefore, this condition results in a more favorable prognosis. DTC CFL also affects patient survival in a complex way. If a strong protective layer is present to prevent the invasion of malignant cells, the longer collagen fibers facilitate immune cells to promptly respond to combat those malignant cells. On the other hand, if the first layer of protection is weak leading to accumulation of a large number of malignant cells in the stromal area, longer collagen fibers may provide a more effective “highway” which assists cancer cell migration. Once the cancer cell attaches to the long collagen fibers, it is easier for them to “grasp” it and subsequently migrate long distances. Figure 5 schematizes the four possible conditions, and survival curves for these groups are presented in Fig. 4.

Furthermore, due to limitations of previous techniques, the quantification of collagen structure and investigation of the relationships between numerical collagen structure parameters and patient survival were not previously feasible. We demonstrated that patients with the best survival (ATC CFD^(+)^ and DTC CFL^(+)^) had increased aggregated collagen area (Fig. 6b) and increased collagen brightness, representing density (Fig. 6c), compared with other groups. However, increases in collagen brightness may have been caused by collagen aggregation. The presence of more collagen in a given small area may also have resulted in a stronger signal. However, in Supp. Fig. 4A, high DTC CFD was observed in patients with the best survival. This suggests that in the DTC area, the collagen fiber was also brighter. Through combining the above data, we conclude that the collagen fiber packing structure differed in such a way that it caused high CFD in both the ATC and DTC regions. Potentially, the collagen fiber packing structure is also associated with the collagen aggregation patterns. In both the DTC and ATC regions, the CFT average value was the same. This further supports the observation that the CFD is not due to a simple clustering of nearby fibers but a real increase of intensity of the fibers (the denser the fibrils within the fibers are, the higher the SHG intensity is, directly linking the intensity of the fiber with the density of fibrils that compose it). As summarized in Supp. Table 3, the ATC is the main actor in the difference between the best and worse survival groups whereas the DTC is the key to highlight differences among all 4 groups and notably the intermediate groups. ATC area ratio is a key parameter to differentiate the best survival patients with the rest of condition, while other parameters contribute to further classify patients on other aspects. Although the results of this study demonstrate that the aggregation of collagen and collagen brightness (represented by CFD and indicating different collagen packing structures) and the DTC CFL are two key collagen-associated parameters that may impact patient survival, the generic mechanism that controls the differences described in our data remains unclear and requires further investigation.

TNBC patients represent a heterogeneous group [47]. Lymph node status is the only prognostic feature for TNBC patients, also supported by our existing data, as shown in Supp. Fig. 5. The use of this prognostic factor is dependent on the detection of cancer cells in the lymph nodes. The percentage and number of patients with positive lymph node status in the four groups is presented in Supp. Fig. 6A and B. In total, 55% of ATC CFD ^(−)^ and DTC CFL^(+)^ patients were lymph node positive, last row in Supp. Fig. 6A; the prognostic model presented in the study shows some correlation with the lymph node status, i.e., 55% of worse survival patients were lymph node positive whereas only 67% of the best survival patients were lymph node negative. Theoretically, it would be possible to build a model based on the collagen parameters investigated in this study to predict patient survival even before metastasis.

The collagen structure parameters extracted in the present study were closely associated with certain pathological parameters, and the mechanism presented in Fig. 5 warrants further investigation. We demonstrated that collagen structure differs in three aspects: (i) the degree of aggregation, (ii) collagen fiber packing structure, and (iii) the length of the collagen in the DTC regions. Although this evidence suggests a potential answer to the historical debate on the role of collagen in breast cancer survival, there may or may not have been causality. Other cellular factors may affect collagen structure, for example, migrating cell behavior, including both immune cell and the migratory capability of cancer cells followed by epithelial-mesenchymal transition (EMT). For example, *NK* cells have antifibrotic properties which decrease with progression of fibrosis [49]. Tumor necrosis factor α (TNF-α) [50], transforming growth factor β (TGF-β) [51–53], IL-11 [54, 55], and OSM [56, 57] are some of the known cytokines involved in the fibrogenesis pathways. However, if any of these factors will impact the abovementioned three aspects and then affect the TNBC patient survival still need further investigation.

Finally, we compared our results with the benign samples and DCIS samples. The data of ATC area ratio, ATC CFD, and DTC CFL are presented in Supp. Fig. 7, 8, 9, respectively. The original data points are presented in panel A, and the mean differences, taking benign as a reference, are presented in panel B. As we mentioned in the “Results” section, ATC CFD^(+)^ and DTC CFL^(+)^ have the best survival. According to the ATC area ratio, ATC CFD, and DTC CFL of these three features, the original data of this best survival population has very similar pattern as patients who were diagnosed as DICS and the mean differences are also very limited. Although other conditions may have one or two parameters closer to DICS patient, they have worse survival as we discussed in Fig. 5.

Although the finding in this work is promising for better stratification of TBNC patients in healthcare system, we must emphasize that it is still preliminary and not yet replicated using whole slide imaging data from an independent cohort. Well-designed rigid validation must be conducted to further confirm its performance before the application in clinical work. The model presented in this work is based on ATC CFD and DTC CFL as highlighted by the blue and green colors in Supp. Table 3; however, other parameters may also contribute to the patient stratification indicating the underline governing mechanism of collagen role in the TNBC patients might be more complicated. It requires further elucidation and is worthy of additional large-scale study. On the other hand, ATC area ratio, highlighted by red in Supp. Table 3, is able to nicely differentiate the best survival patients with other three groups. In practical, this more simplified model can possibly provide some immediate insight in clinic. Tumor progression is an outcome of mutations in multiple different genes, and the relationships between collagen characteristics and other critical tumor compartments, such as the immune response, EMT, adipose, and angiogenesis, should be investigated in a more systematic study.

## Supplementary information

**Additional file 1: Supplementary Figure 1**. Collagen length and density in comparison with tumour size and grades. **Supplementary Figure 2**. Kaplan Meier survival curves for Two individual parameters of prognostic value. **Supplementary Figure 3**. Kaplan Meier survival curves for tumour size and grade. **Supplementary Figure 4**. Some other key aspects on the collagen structural differences in the four given groups. **Supplementary Figure 5**. Kaplan Meier Survival curves for the Lymph node status. **Supplementary Figure 6**. Lymph node status(+/-) distributions in the four groups of patients. **Supplementary Figure 7**. Comparison of the ATC Area Ratio between the four groups of patients, Benign samples and the DCIS patient cohort. **Supplementary Figure 8**. Comparison of the ATC CFD between the four groups of patients, Benign samples and the DCIS patient cohort. **Supplementary Figure 9**. Comparison of the DTC CFL between the four groups of patients, Benign samples and the DCIS patient cohort. **Supplementary Table 1**. Comprehensive Parameters List measured in SHG/TPE images for TNBC patient cohort. **Supplementary Table 2**. Key Parameters List and their definition for TNBC patient cohort. **Supplementary Table 3**. Pairwise *P*-values for the set of features measured in 4 groups.

## Data Availability

The images and numerical data of this work will be available upon request.
