# Supplementary material for: Quantitative stain-free imaging and digital profiling of collagen structure reveal diverse survival of triple negative breast cancer patients
Source: Breast Cancer Res. 2020 May 6;22:42. doi: 10.1186/s13058-020-01282-x (PMC7204022; doi:10.1186/s13058-020-01282-x)

**
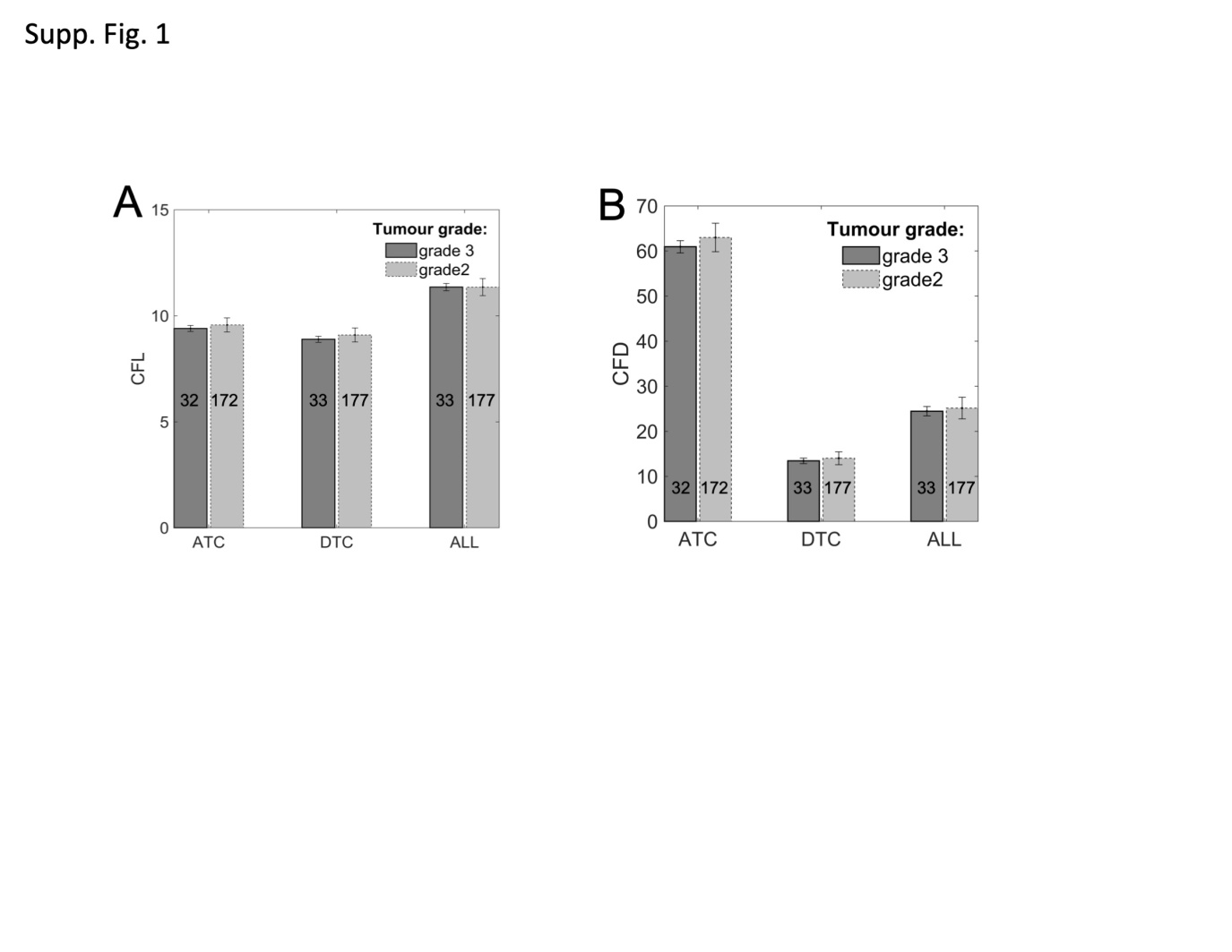
Supplementary Figure 1.** **Collagen length and density in comparison with tumour size and grades.** (**A**). ATC CFL and DTC CFL are not associated with tumour grade. (**B**) CFD showed no association with tumour grades. (Error bars represent SE of the mean. *p<0.05, **p<0.0, ***p<0.001 and >***p<0.0001, with comparisons indicated by lines. P values calculated using one way ANOVA analysis and tukey’s HSD test.)

**
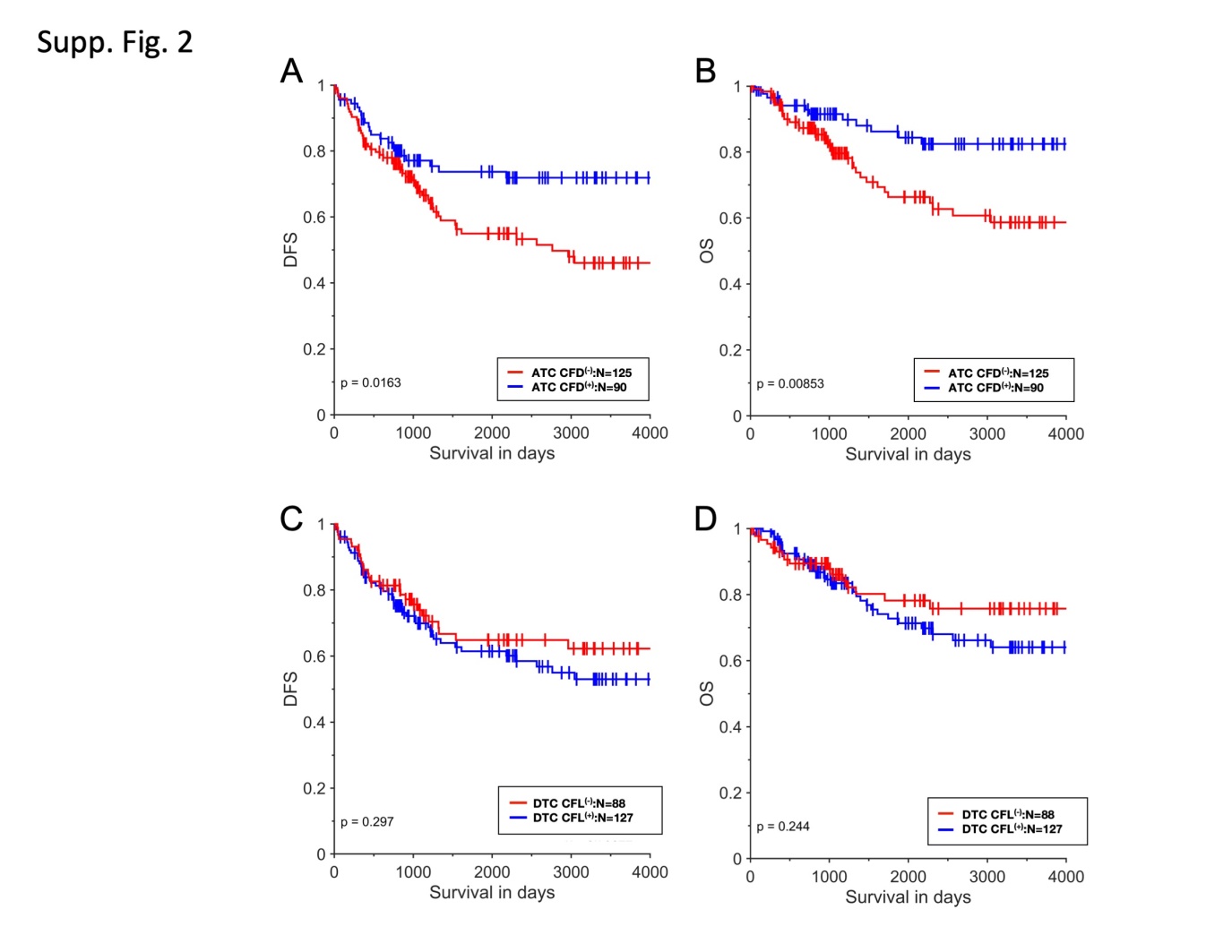
Supplementary Figure 2. Kaplan Meier survival curves for Two individual parameters of prognostic value**. ATC CFD is one of the prognostic parameters impacting **(A)** DFS and **(B)** OS, respectively. DTC CFL is another prognostic parameter affecting **(C)** DFS and **(D)** OS, respectively. The cut off values of ATC CFD and DTC CFL were determined according *t* statistical analysis, as detailed in the Methods section.


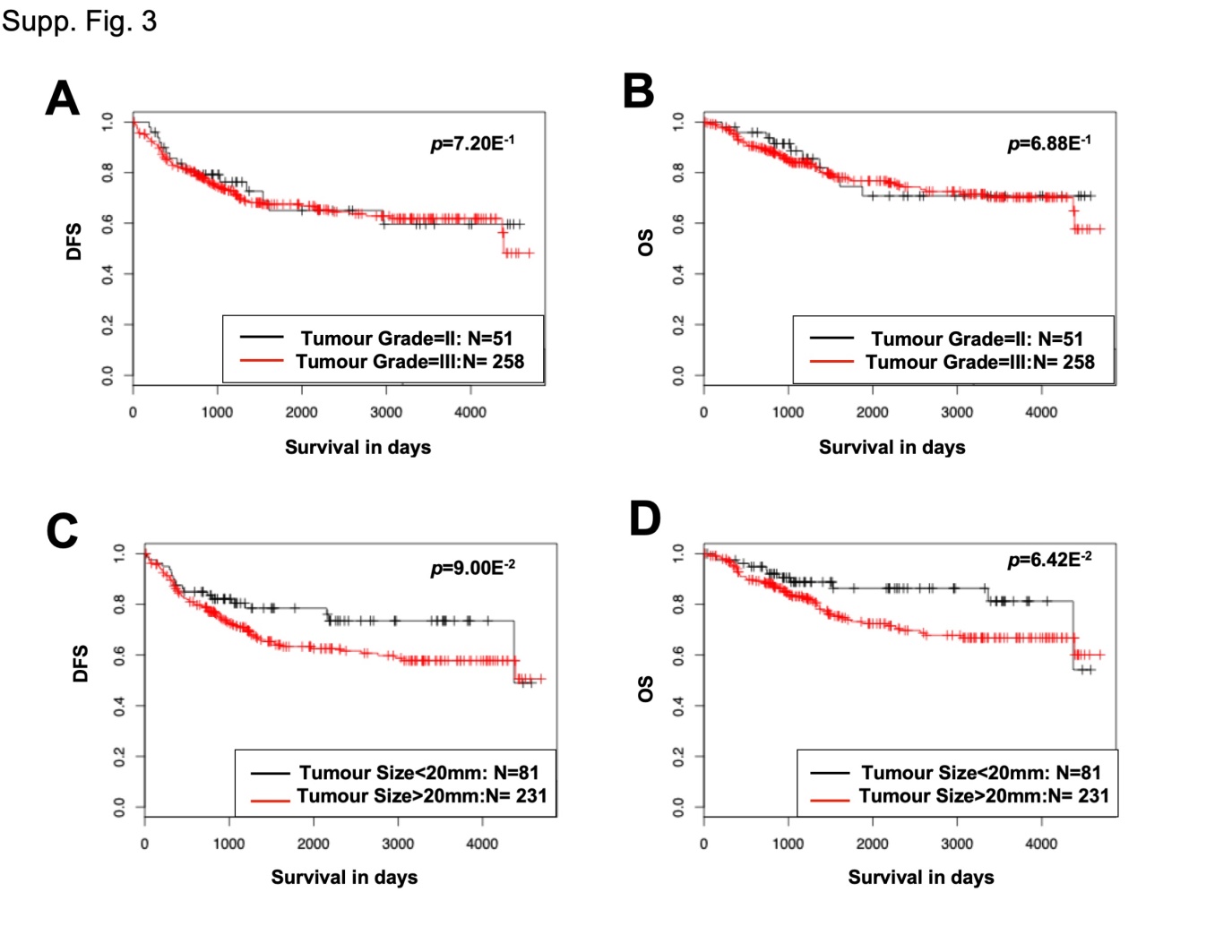
**Supplementary Figure 3.** **Kaplan Meier survival curves for tumour size and grade.**  **(A-B)** Tumour grade and **(C-D)** tumour size are not useful prognostic parameters for TNBC patients in this series.

**
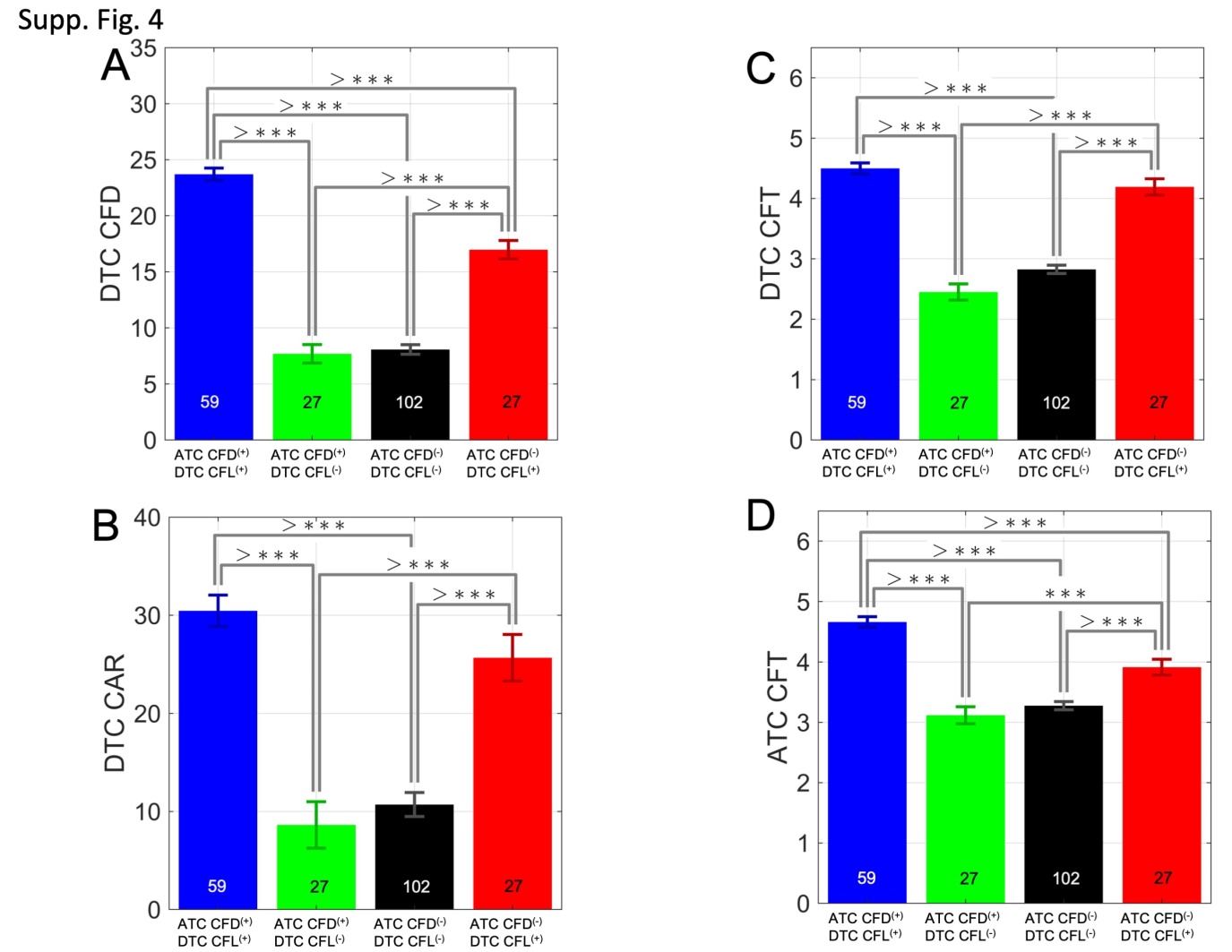
**

**Supplementary Figure 4.** **Some other key aspects on the collagen structural differences in the four given groups**. **(A).** The DTC CFD in ATC CFD^(+)^ and DTC CFL^(+)^ are higher than other groups, while its DTC CAR, as shown in **(B)**, is not significantly different than ATC CFD^(-)^ and DTC CFL^(+)^ patients. This is indirect proof that stronger ATC CFD reading in Fig. 6C is not due to collagen aggregation. The DTC CFT and ATC CFT also show a significant difference between the four groups of patients. (Error bars represent SE of the mean. *p<0.05, **p<0.0, ***p<0.001 and >***p<0.0001, with comparisons indicated by lines. P values calculated using one way ANOVA analysis and tukey’s HSD test.)

**
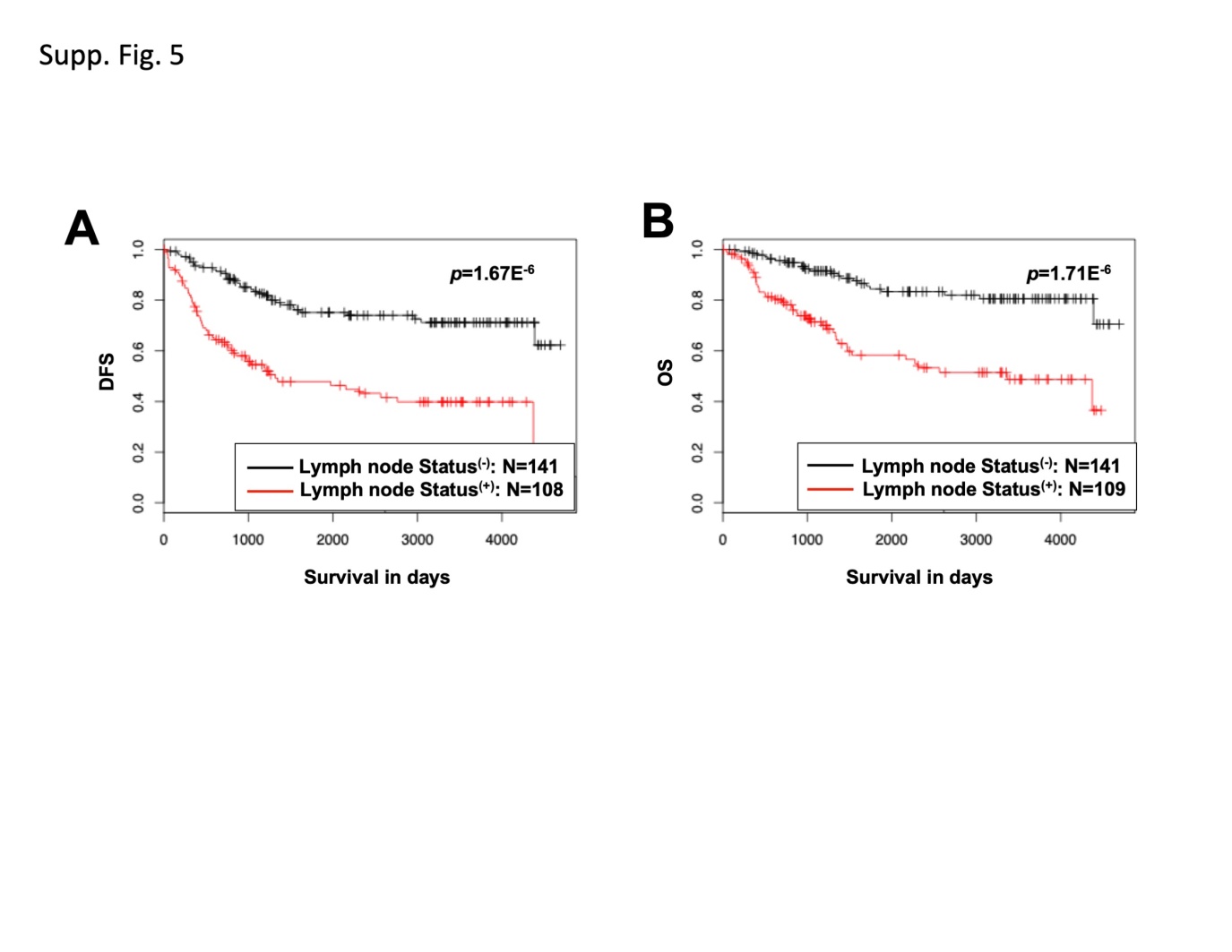
Supplementary Figure 5. Kaplan Meier Survival curves for the Lymph node status.** It is currently the only strong prognostic parameter for TNBC, based on our data.

**
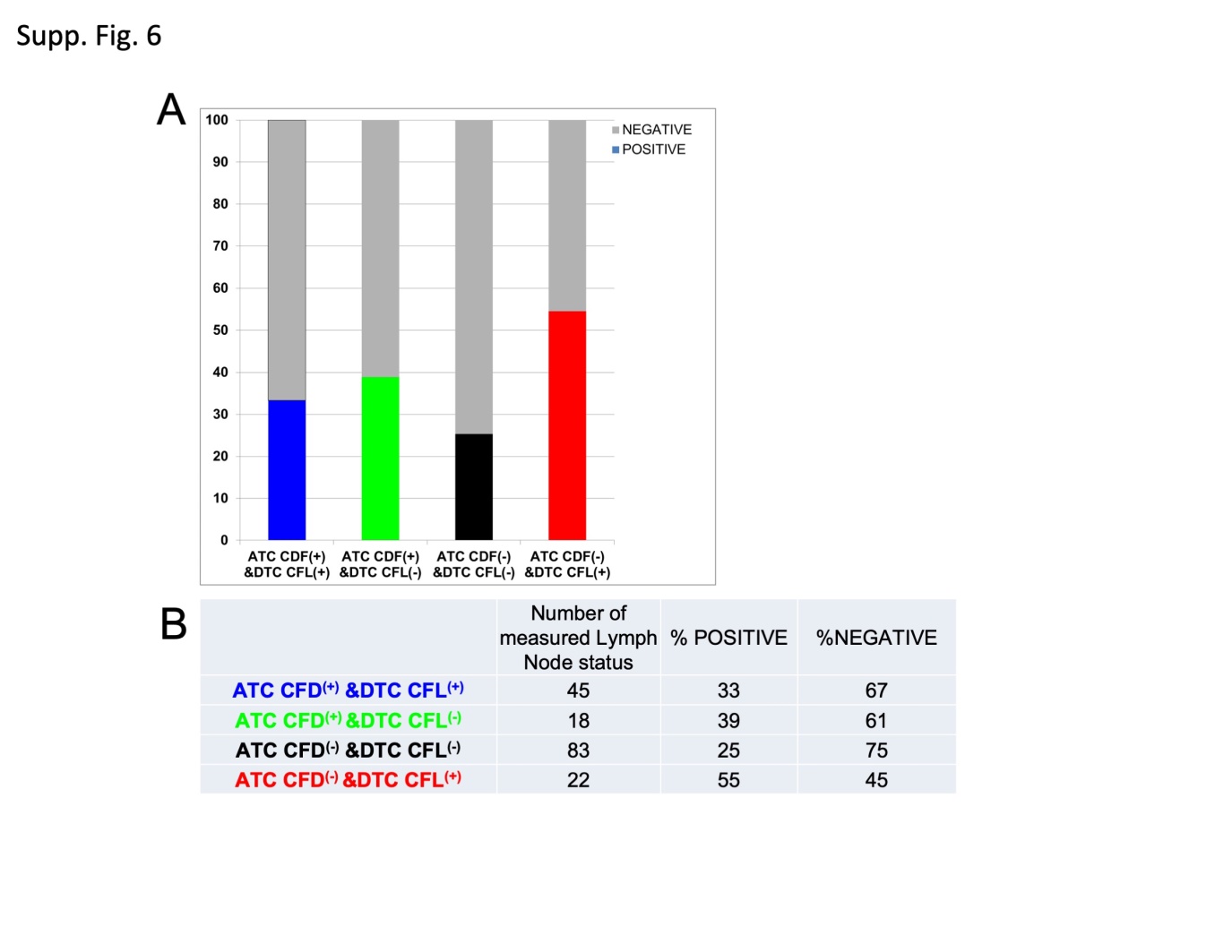
Supplementary Figure 6. Lymph node status^(+/-)^ distributions in the four groups of patients**. **(A)** The distribution of lymph node status^(+/-)^ in our groups. **(B)** The number of cases. Our prognostic model is different from lymph node status. Compared with Fig. 6D, DTC CFL^(+)^ is a potential predictor of higher lymph node status^(+)^ in ATC CFD^(-)^ & DTC CFL^(+)^ and ATC CFD^(+)^ and DTC CFL^(+)^, i.e. the second and third column in **(A)**. Theoretically, it would also be possible to use collagen structural information to build a model to predict lymph node status, which would have strong clinical value in terms of patient management.

**
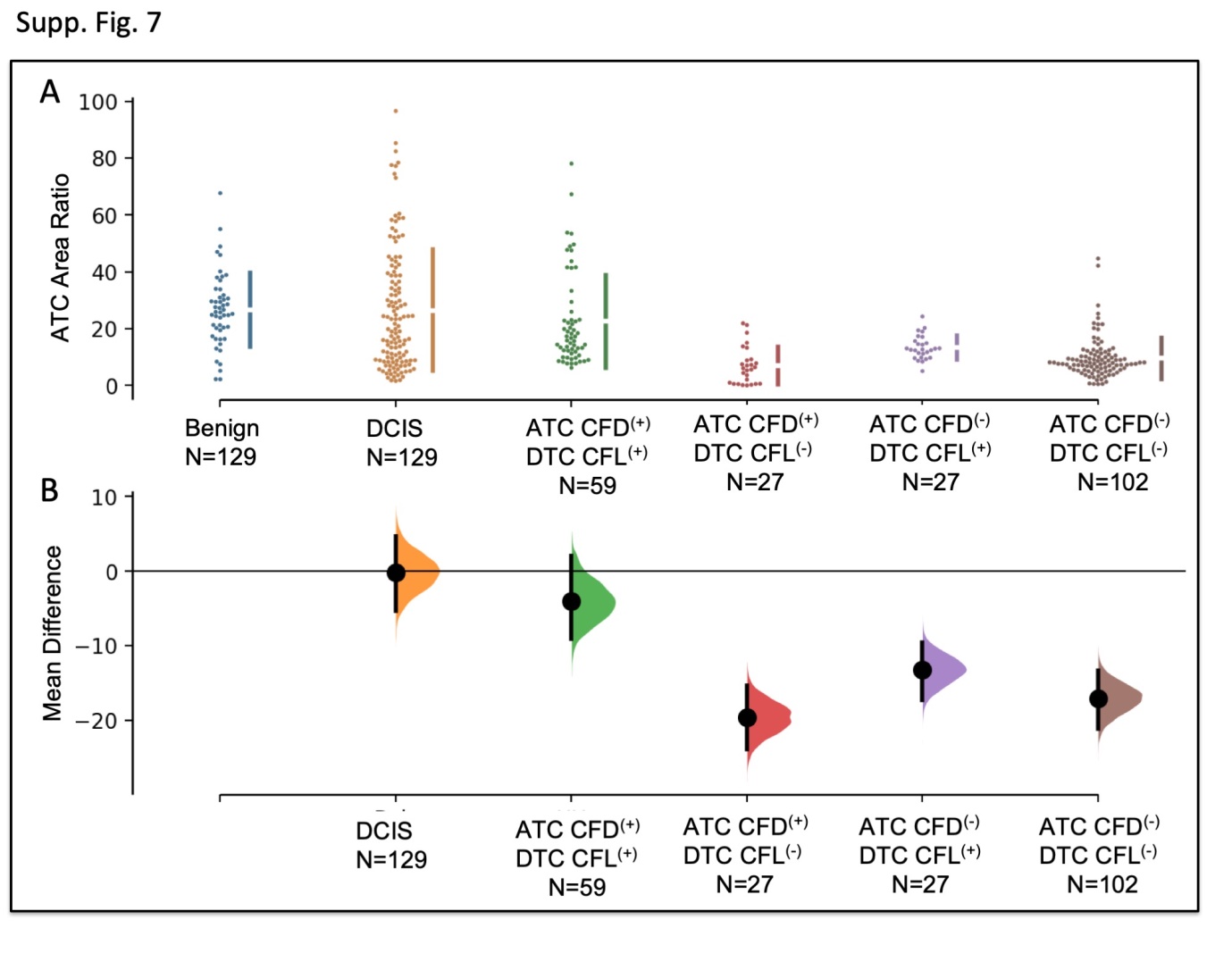
**

**Supplementary Figure 7. Comparison of the ATC Area Ratio between the four groups of patients, Benign samples and the DCIS patient cohort.**

**
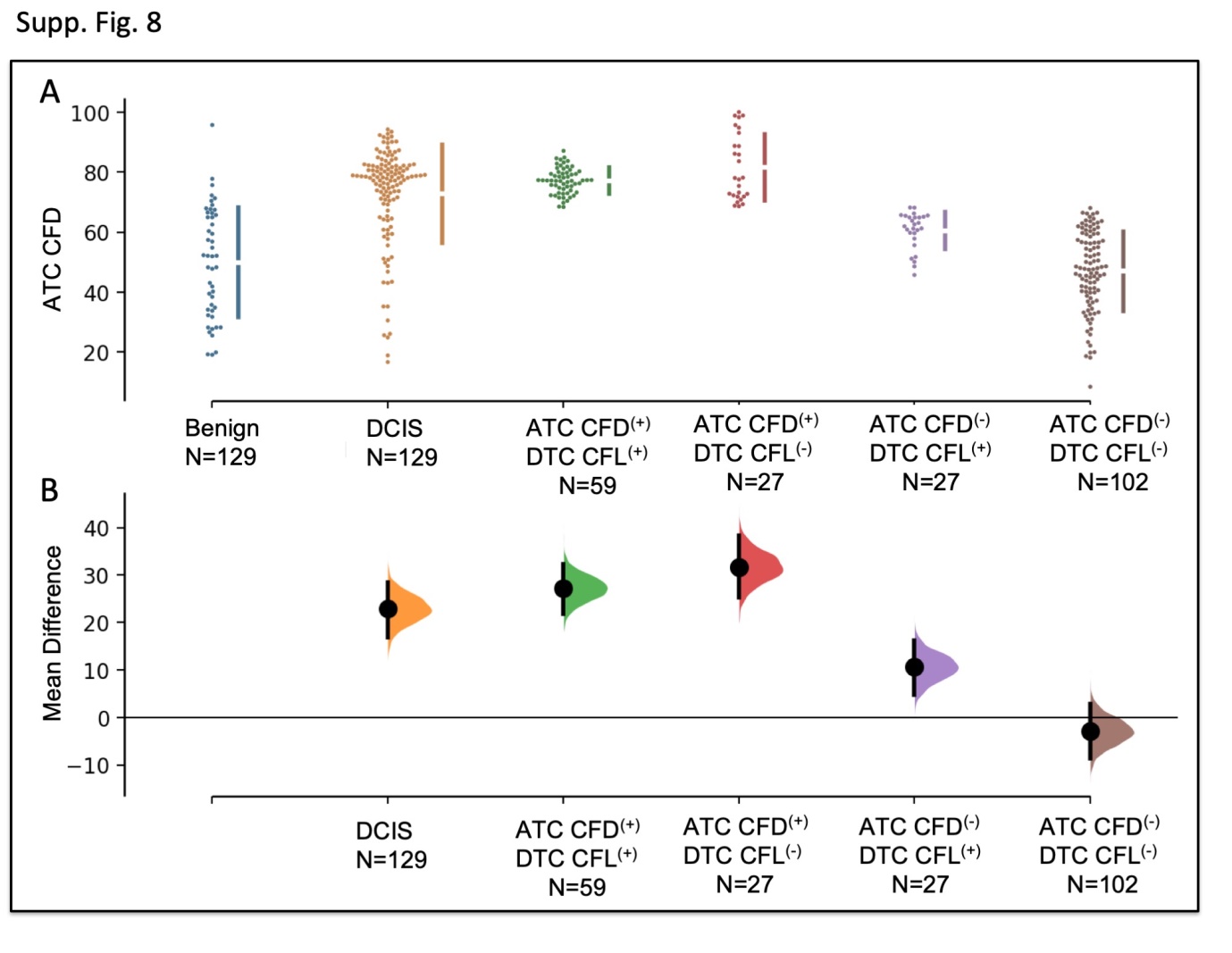
**

**Supplementary Figure 8. Comparison of the ATC CFD between the four groups of patients, Benign samples and the DCIS patient cohort.**

**
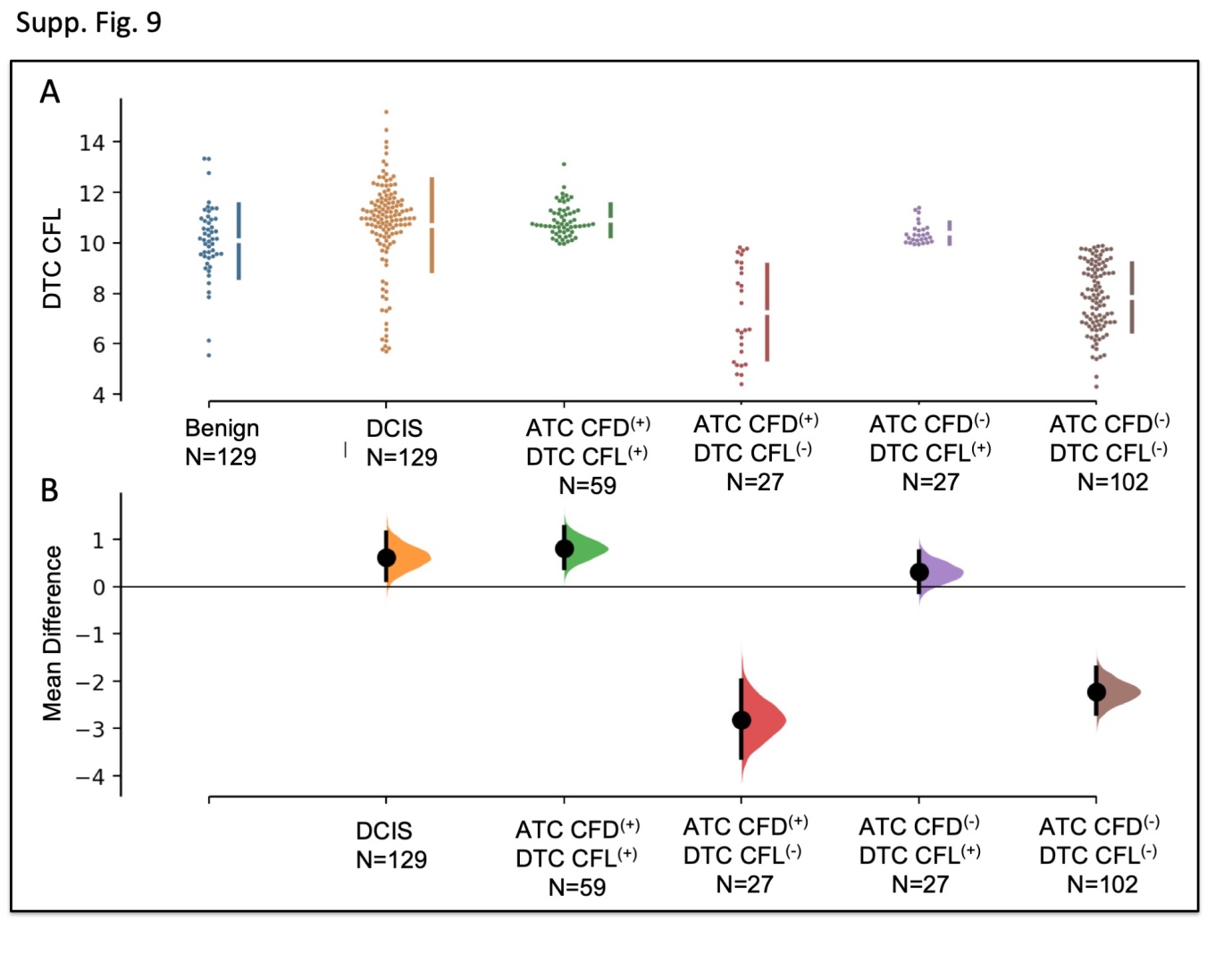
**

**Supplementary Figure 9. Comparison of the DTC CFL between the four groups of patients, Benign samples and the DCIS patient cohort.**

**Supplementary Table 1. Comprehensive Parameters List measured in SHG/TPE images for TNBC patient cohort.**

**
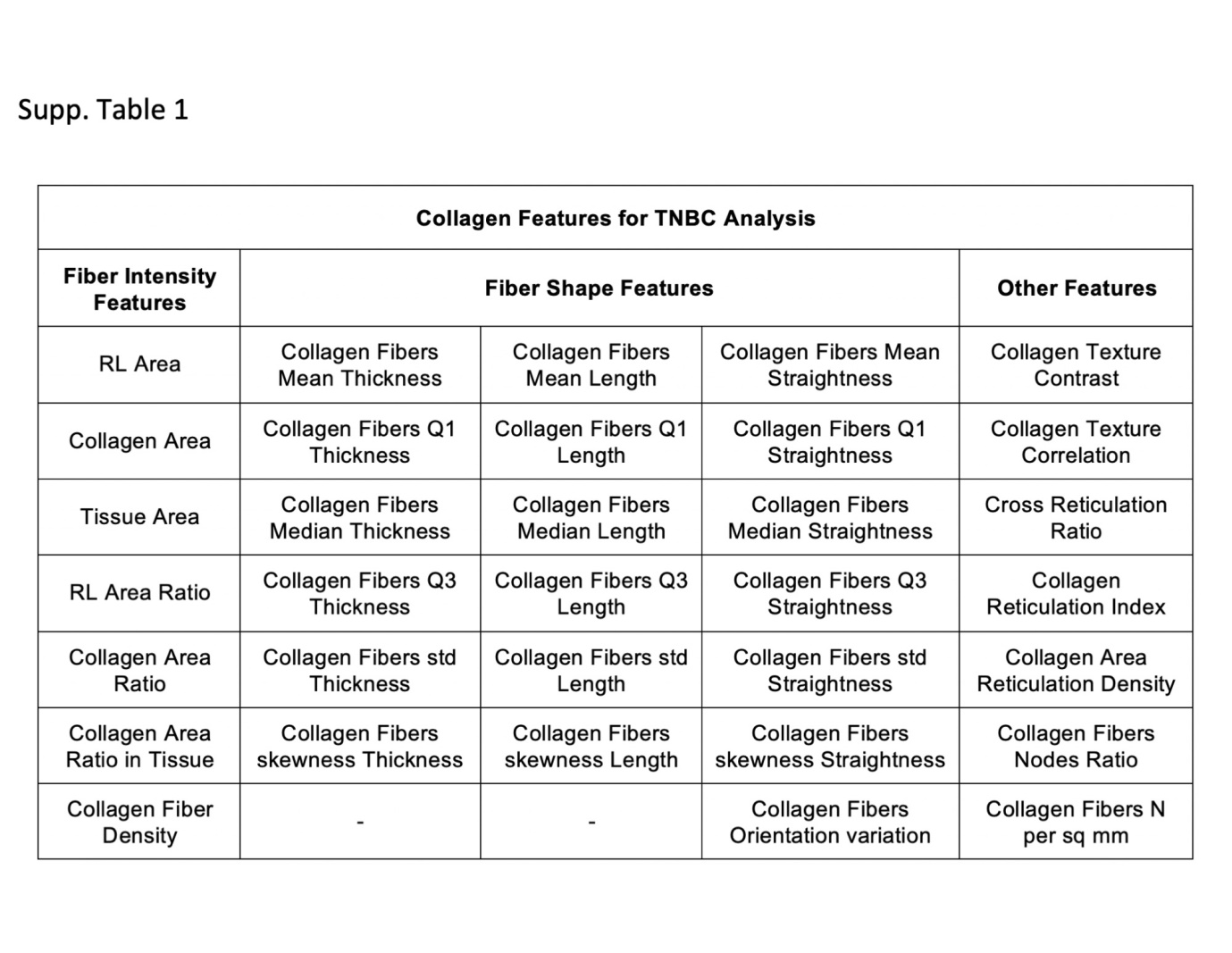
**

**Supplementary Table 2. Key Parameters List and their definition for TNBC patient cohort.**

**
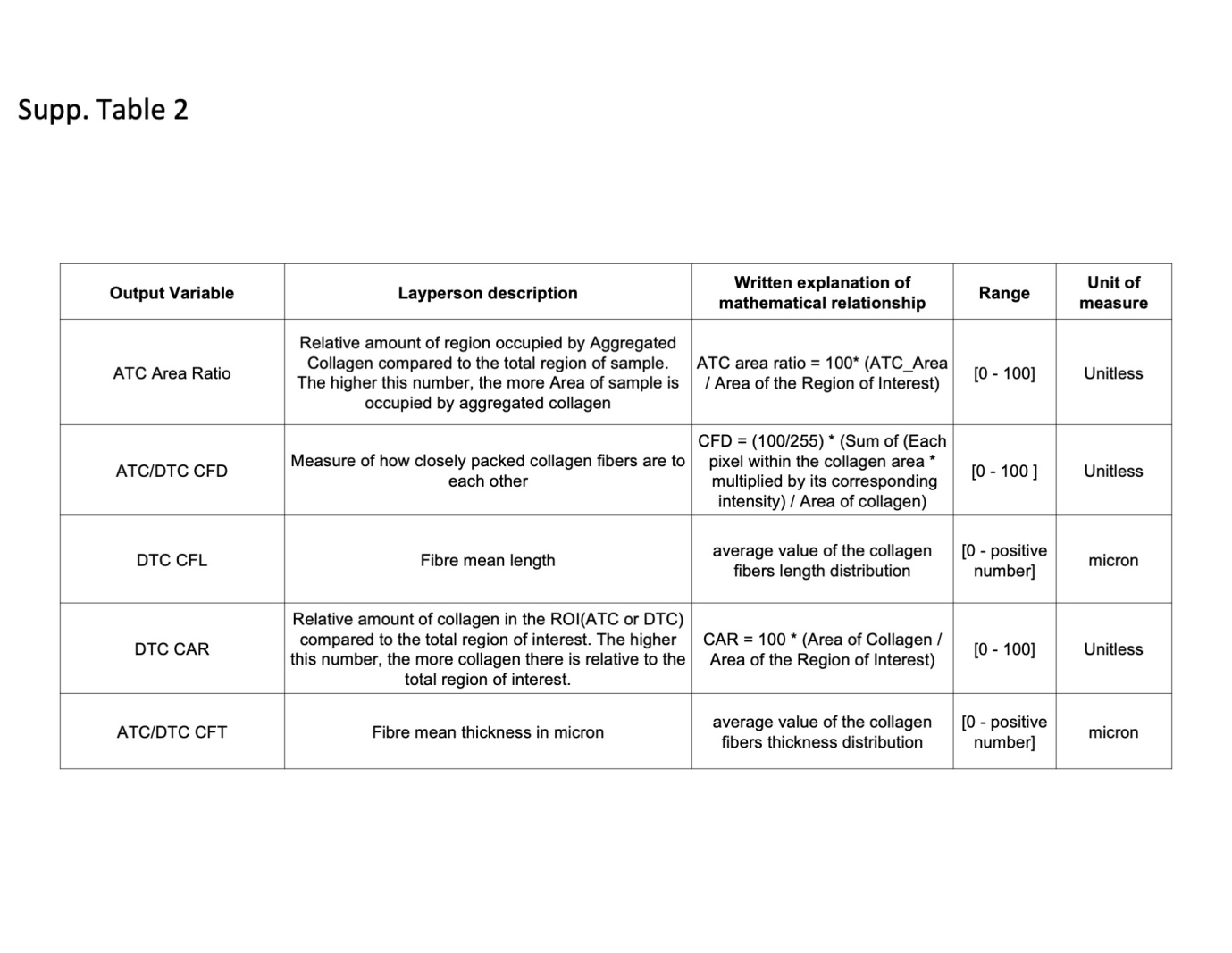
**

**Supplementary Table 3.** **Pairwise P-values for the set of features measured in 4 groups.** Groups 1 to 4 from best survival to worse survival respectively (see Fig.4). This table highlights which features are key for differentiating different groups of patient.


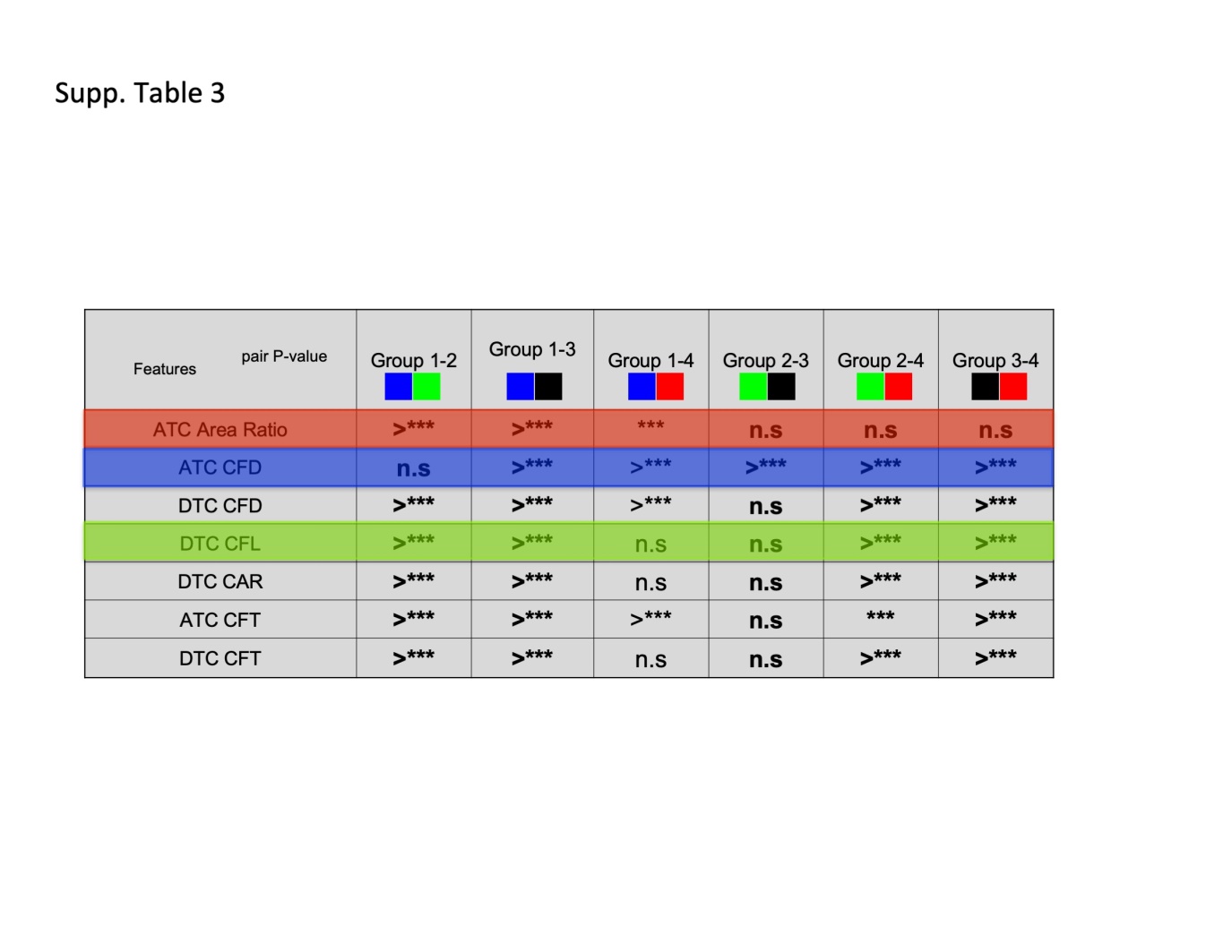

Supplement: Supplementary file 1 — Additional file 1: Supplementary Figure 1. Collagen length and density in comparison with tumour size and grades. Supplementary Figure 2. Kaplan Meier survival curves for Two individual parameters of prognostic value. Supplementary Figure 3. Kaplan Meier survival curves for tumour size and grade. Supplementary Figure 4. Some other key aspects on the collagen structural differences in the four given groups. Supplementary Figure 5. Kaplan Meier Survival curves for the Lymph node status. Supplementary Figure 6. Lymph node status(+/-) distributions in the four groups of patients. Supplementary Figure 7. Comparison of the ATC Area Ratio between the four groups of patients, Benign samples and the DCIS patient cohort. Supplementary Figure 8. Comparison of the ATC CFD between the four groups of patients, Benign samples and the DCIS patient cohort. Supplementary Figure 9. Comparison of the DTC CFL between the four groups of patients, Benign samples and the DCIS patient cohort. Supplementary Table 1. Comprehensive Parameters List measured in SHG/TPE images for TNBC patient cohort. Supplementary Table 2. Key Parameters List and their definition for TNBC patient cohort. Supplementary Table 3. Pairwise P-values for the set of features measured in 4 groups. [file 13058_2020_1282_MOESM1_ESM.docx]
